# How can patient registries facilitate guideline-based healthcare? A retrospective analysis of the CEDATA-GPGE registry for pediatric inflammatory bowel disease

**DOI:** 10.1186/s12913-023-09639-6

**Published:** 2023-06-17

**Authors:** M. Leiz, M. Knorr, K. Moon, L. Tischler, K. Sohrabi, S. Cantez, J. Däbritz, J. de Laffolie, N. van den Berg, CEDATA GPGE Study Group, S. Buderus, P. Bufler, S. Dammann, A. Hauer, K.-M. Keller, A. Krahl, M. Laaß, T. Lang, C. Posovszky, B. Rodeck, S. Trenkel

**Affiliations:** 1 Institute for Community Medicine, University Medicine, Greifswald, Germany; 2 Technical University of Applied Sciences, Giessen, Germany; 3 General Pediatrics & Neonatology, Justus-Liebig-University, Giessen, Germany; 4 Department of Pediatrics, Greifswald University Medical Center, Greifswald, Germany

**Keywords:** Pediatric inflammatory bowel disease (PIBD), Registry, Treatment guidelines

## Abstract

**Background:**

Early diagnosis is mandatory for the medical care of children and adolescents with pediatric-onset inflammatory bowel disease (PIBD). International guidelines (‘Porto criteria’) of the European Society for Pediatric Gastroenterology, Hepatology and Nutrition recommend medical diagnostic procedures in PIBD. Since 2004, German and Austrian pediatric gastroenterologists document diagnostic and treatment data in the patient registry CEDATA-GPGE on a voluntary basis. The aim of this retrospective study was to analyze whether the registry CEDATA-GPGE reflects the Porto criteria and to what extent diagnostic measures of PIBD according to the Porto criteria are documented.

**Methods:**

Data of CEDATA-GPGE were analyzed for the period January 2014 to December 2018. Variables representing the Porto criteria for initial diagnostic were identified and categorized. The average of the number of measures documented in each category was calculated for the diagnoses CD, UC, and IBD-U. Differences between the diagnoses were tested by Chi-square test. Data on possible differences between data documented in the registry and diagnostic procedures that were actually performed were obtained via a sample survey.

**Results:**

There were 547 patients included in the analysis. The median age of patients with incident CD (*n* = 289) was 13.6 years (IQR: 11.2–15.2), of patients with UC (*n* = 212) 13.1 years (IQR: 10.4–14.8) and of patients with IBD-U (*n* = 46) 12.2 years (IQR: 8.6–14.7).

The variables identified in the registry fully reflect the recommendations by the Porto criteria. Only the disease activity indices PUCAI and PCDAI were not directly provided by participants but calculated from obtained data. The category ‘Case history’ were documented for the largest part (78.0%), the category ‘Imaging of the small bowel’ were documented least frequently (39.1%). In patients with CD, the categories ‘Imaging of the small bowel’ (χ^2^ = 20.7, Cramer-V = 0.2, *p* < 0.001) and ‘Puberty stage’ (χ^2^ = 9.8, Cramer-V = 0.1, *p* < 0.05) were documented more often than in patients with UC and IBD-U.

**Conclusion:**

The registry fully reproduces the guideline’s recommendations for the initial diagnosis of PIBD. The proportion of documented diagnostic examinations varied within the diagnostic categories and between the diagnoses. Despite technological innovations, time and personnel capacities at participating centers and study center are necessary to ensure reliable data entry and to enable researchers to derive important insights into guideline-based care.

**Supplementary Information:**

The online version contains supplementary material available at 10.1186/s12913-023-09639-6.

## Introduction

Long-term data collection is irreplaceable to gather important information about diseases [1], e.g. inflammatory bowel diseases (IBD). IBD includes Crohn's disease (CD), ulcerative colitis (UC) and unclassified inflammatory bowel disease (IBD-U). 19–25% of newly diagnosed IBD patients are under the age of 20 years [2, 3]. The incidence and prevalence of pediatric inflammatory bowel diseases (PIBD) increased in recent years, especially in industrial countries [4–6], but also in developing countries [7]. An analysis of data from a German statutory health insurance in 2017 found an incidence for CD of 10.3 and for UC of 6.0 per 100,000 insured children and adolescents under the age of 18 [8]. The federal health reporting system in Germany reported 15 newly diagnosed CD- and 18 UC-cases per 100,000 inhabitants under the age of 15 in 2017 [9].

PIBD is associated with impaired quality of life, longtime morbidity and mortality, delayed physical development, impairment of social life and mental health burden [10]. An early age at diagnosis is also associated with a higher burden of lifelong costs of health care [11]. Early diagnosis is mandatory to the physical, psychological and social development of pediatric patients.

PIBD is characterized by intestinal and extraintestinal symptoms, e.g. abdominal pain, diarrhea or blood in stool, but also growth retardation, joint and skin involvement, fever of unknown origin, liver disease and anorexia which warrants a wide set of differential diagnosis and needs to be coherently investigated [12, 13]. A significant diagnostic delay is associated with a higher risk of growth failure in CD and of more extensive disease in UC [14–16]. The IBD working group of the European Society of Pediatric Gastroenterology, Hepatology and Nutrition (ESPGHAN) met in 2014 in Porto and developed guidelines for the diagnostic procedure in pediatric patients with IBD (‘Porto criteria’) [17, 18].

**Fig. 1:**
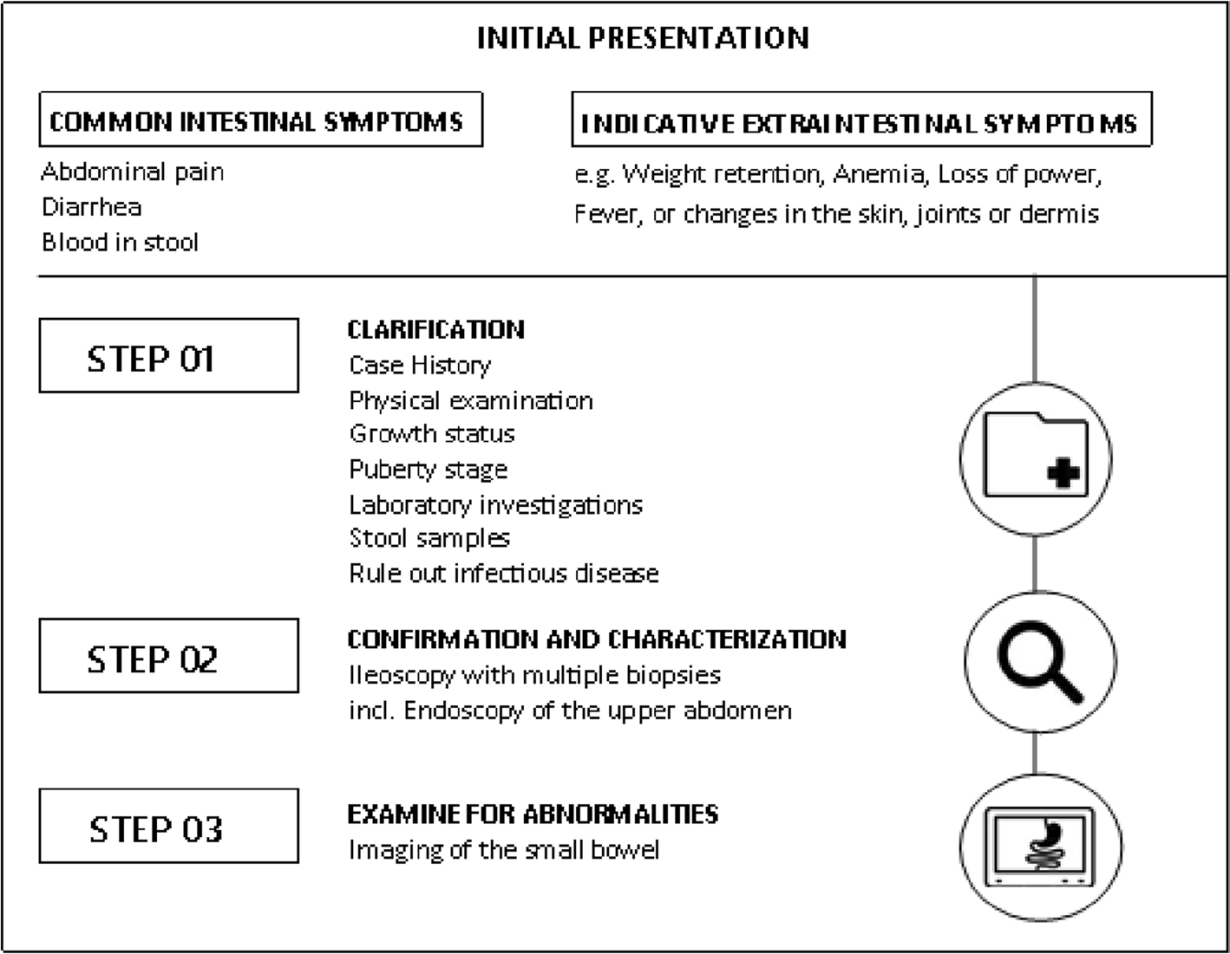
Steps for guideline-compliant diagnosis of PIBD considering the Porto criteria. Own presentation based on Buderus et al. [13] and Däbritz et al. [19]

Figure 1 shows the most important steps towards a confirmed diagnosis of PIBD, considering the Porto criteria. In the first step, when PIBD is suspected, a thorough medical history (including family history), laboratory tests and a physical examination of the patient should be prioritized. Subsequently, in the second step, endoscopic and histological examinations should be performed for all patients to confirm the diagnosis and to further characterize the subtype of IBD. Finally, the process is completed by imaging of the small bowel [13, 17].

The literature reports great variability in the diagnostic measures documented in diagnosing new patients with PIBD [10, 20]. As part of an initiative to improve quality in health care in 2007 it was shown that only 47.2% (weighted adherence) of children received recommended diagnostic measures [21]. A survey of clinicians from the ESPGHAN PIBD Working Group on the use and application of the Porto criteria in 2019 found that 80% of the responding centers (*n* = 106, response: 65.4%) met all recommended Porto criteria in at least 80% of their patients (self-reported by treating physicians) [22]. Participation in the registry of the collaborative network ‘Improve care now’ in the US led to a significantly higher documentation of adequate guideline-based care during outpatient visits [20, 23].

Since 2004, German and Austrian pediatric gastroenterologists can document diagnostic and treatment data of their patients with IBD in the patient registry CEDATA-GPGE. Regular participation in the registry can increase awareness and adherence to guidelines over the years. A previous study with data from the CEDATA-GPGE registry shows an increase in the frequency of relevant instrumental diagnostics over a 6-year period (2004–2010) [24].

The aim of this study was to investigate the extent of diagnostic measures of PIBD documented in the patient registry CEDATA-GPGE during a time of technological change and innovation in the registry with a newly implemented online platform. Our research questions were:

1. Which Porto criteria for diagnostics are represented in CEDATA-GPGE?
2. To what extent are the Porto criteria for diagnostic measures documented in the registry for the years 2014–2018?
3. What implications can the results provide for health care research based on patient registries?

This investigation was conducted as part of the German innovation fund project ‘CED-KQN Big Data—eHealth: Improving the health care of children and adolescents with inflammatory bowel diseases’.

## Material and methods

### CEDATA-GPGE registry

The German-speaking Society of Pediatric Gastroenterology and Nutrition (GPGE) initiated the CEDATA-GPGE registry in 2004. It collects clinical and paraclinical data of children and adolescents with IBD in German-speaking countries. Participation and documentation in the registry are voluntary and mainly carried out by certified gastroenterological centers for pediatric patients in Germany and Austria. Only pediatric gastroenterologists document in the registry. Non-pediatric gastroenterologists (e.g. internist gastroenterologists) are not actively recruited.

Since the start of the registry, data of more than 6,000 children and adolescents were included into the registry with over 50,000 documented patient contacts [25]. From 2004 to 2010, documentation was paper-based and postal with central data input. Between July 2010 and July 2013 no new patients were included because of logistical and financial reasons and reconstruction of the platform. Since August 2013, documentation can be conducted via an internet-based platform. In December 2016, the CEDATA-GPGE registry was transferred to the Online-Tool 2.0. The resulting web application allows navigation through individual documentation forms, displaying only fields that are relevant in the current context. The user is supported during the documentation process by the transfer of various field entries. The data is subjected to an automatic real-time plausibility check upon data input. Due to the introduction of new fields, the data structures have been redefined, making more complex evaluations possible [26].

The initial documentation form (see Additional file 1) contains information about the case history, symptoms at presentation, performed diagnostics and initial therapy. Ideally, the follow-up data (therapy, complications, operations, changes in condition of patients) is documented at least twice a year (see Additional file 1). More documentation can be provided, depending on the number of contacts of the patients with the treating physicians.

Personal information of the patients is separated from medical data and transmitted directly by the registration centers to an independent trust center in Berlin. The identifying data are stored separately from medical records in accordance with the provisions of the Data Protection Act. The study center of the Department of General Pediatrics and Neonatology of the Justus-Liebig-University Giessen administers the patient registry CEDATA-GPGE.

### Patients

We analyzed data of patients that were included in CEDATA-GPGE between 01 January 2014 and 31 December 2018, during the first five years of the online platform by participating pediatric gastroenterological centers.

Patients that had no documentation within 90 days after the date of diagnosis were excluded. Additionally, patients recruited before the start of the registry’s online data entry tool 2.0 on 16 December 2016 and that had no documentation later than 14 days after diagnosis were excluded.

### Participating centers

Information on the organizational and medical structures of the centers were taken from the homepage of the GPGE and the online available annual quality reports of the hospitals, which have been mandatory for publication in Germany since 2005. In Austria such a reporting system did not exist for our observation period. In the consequence, no structural data were available for Austrian centers.

### Porto criteria

The international recommendations (Porto criteria) of the European Society for Pediatric Gastroenterology, Hepatology and Nutrition (ESPGHAN) are the evidence base for medical diagnostic examinations and therapeutic decisions in Germany and Austria. According to these criteria for diagnosis, the following diagnostic work up should be performed to confirm the diagnosis of PIBD (Table 1).

**Table 1:** Recommended diagnostic work up for a confirmed diagnosis of PIBD (Porto criteria). Own presentation based on Levine et al. [17] and Turner et al. [27]

Recommended diagnostic work up by ESPGHAN
Case history
Physical examination
Growth status
Nutrition status
Puberty stage
Ileocolonoscopy with multiple biopsies
Initial laboratory investigations
Stool cultures from at least 3 independent stool samples
Endoscopy of the upper gastrointestinal tract (Esophagogastroduodenoscopy)
Imaging of the small bowel
Disease activity
Diagnosis by gastroenterologist with pediatric expertise
Children under 2 years: additional immunological and allergy tests

Children up to the age of six are subsumed under the term ‘very early onset-IBD’ (VEO-IBD). Patients at this age group should be screened for immunodeficiencies, because undetected immunodeficiencies can lead to severe complications in therapy [13]. According to the Porto criteria, immunological examinations and allergy tests are especially recommended for children under two years of age [17].

### Analysis

#### Identification of guideline-equivalent variables

By screening the variables of the initial documentation form of CEDATA-GPGE, we identified the variables that represent the Porto criteria for initial diagnostic. The identified variables were grouped into 12 categories. Since testing for immunodeficiencies mainly applies to patients with VEO-IBD (age ≤ 6 years), and is also useful in situations with a difficult course and atypical accompanying symptoms, these parameters were considered separately.

#### Documented initial diagnostic work up

The continuous and discrete variables of the registry were recoded to dichotomous variables: variables that contained a value according to the established scaling of the registry were recoded to 1 (= documented). Variables that did not contain a value (= missings) or that were not specified were marked with 0 (= not documented).

Our aim was to show how many examinations for each diagnostic category were documented on average in proportion to the maximum possible amount. Therefore, we calculated the average of the measures documented in each category for the diagnoses CD, UC, and IBD-U.

Furthermore, we calculated the pediatric Crohn´s disease activity index (PCDAI) and the pediatric ulcerative colitis activity index (PUCAI) by using validated algorithms [28, 29].

Due to the high relevance of the instrumental diagnostics for a confirmed diagnosis of PIBD, which is represented by the categories ‘Endoscopy of the upper abdomen’, ‘Ileocolonoscopy with multiple biopsies’ and ‘Imaging of the small bowel’, the percentage of performance per year and diagnosis was calculated.

Differences in diagnostic measures between the diagnoses CD, UC and IBD-U were tested by Chi-square test. All analyzed patient data were pseudonymized. Data processing and statistical calculations were performed with IBM SPSS Statistics Version 27.0 (by IBM Corp.©, Armonk, New York, USA).

### Supplementary survey

The aim of this supplementary survey was to identify differences between the data documented in the registry and the screenings actually performed, i.e., whether there may be an underreporting. Based on the results on center structures using the quality reports, the five largest centers were contacted by email with a request to give information on the following questions: number of treated patients with PIBD; proportion of under 6-year-olds screened for immunodeficiencies and proportion of under 2-year-olds screened for immunodeficiencies and allergies; proportion of patients with PIBD reported in the CEDATA-GPGE registry; if not all patients are reported in the registry, reasons for that.

### Ethics and informed consent

Data assessment and analyses were performed in accordance with the guidelines and recommendations for Good Epidemiological Practice [30] and in accordance with the Declaration of Helsinki [31]. The registry CEDATA-GPGE with recruiting and documentation procedures and scientific analysis were approved by the Ethics Committee of the Justus-Liebig University Giessen (ethic approval protocol number 07/11) and by all ethics committees of centers involved. Participating centers from Austria have an additional local ethics vote. In order for patients to be included in the registry, they or their legal guardians must be fully informed about the aim and purpose of the registry and give their written consent [32].

## Results

### Study sample

**Fig. 2:**
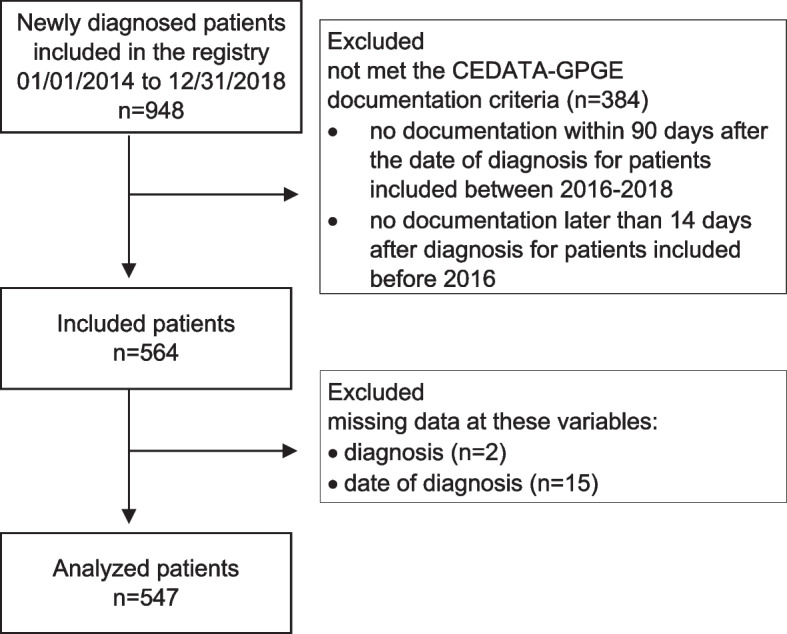
Flowchart of included and analyzed patients

Of 547 included patients (Fig. 2), 289 (52.8%) patients were newly diagnosed with CD, 212 (38.8%) with UC and 46 (8.4%) with IBD-U.

The median age in patients with CD was 13.6 years (interquartile range (IQR): 11.2–15.2), in patients with UC 13.1 years (IQR: 10.4–14.8) and in patients with IBD-U 12.2 years (IQR: 8.6–14.7). In patients with CD, 42.9% (*n* = 124) were female, in UC 46.7% (*n* = 99) and in IBD-U 45.7% (*n* = 21).

The typical triad of symptoms in CD were the most frequently documented symptoms of the patients in our study population: diarrhea (66.1%), abdominal pain (75.8%), and weight stagnation/weight loss (56.7%). In UC and IBD-U, diarrhea (UC: 78,3%, IBD-U: 69,6%), abdominal pain (UC: 71.7%, IBD-U: 65.2%), and blood in stool (UC: 81.1%, IBD-U: 69,6%) were the three most common symptoms. In the overall study population, these were also the three most common symptoms: abdominal pain (73.3%, *n* = 401), diarrhea (71.1%, *n* = 389) and visible blood in stool (57.0%, *n* = 312).

In 22% (*n* = 119) of the patients, a family member had been previously diagnosed with IBD. Of these, 13.5% (*n* = 73) had CD, 7.4% (*n* = 40) had UC and 1.1% (*n* = 6) had IBD-U.

Patients were enrolled by 33 centers in Germany (*n* = 28) and Austria (*n* = 5). Among them, there were 9 university hospitals and 22 teaching hospitals. 21 centers (63.6%) were certified by GPGE (Germany: 19, Austria: 2).

85% (*n* = 467) of all patients were newly diagnosed by a pediatric gastroenterologist, 11.2% (*n* = 61) by an adult gastroenterologist and 0.9% (*n* = 5) by a pediatric surgeon (missings: *n* = 14).

### Identification of variables according to the guidelines in CEDTA-GPGE

We identified 41 diagnostic variables from the CEDATA-GPGE registry. These variables correspond to the Porto criteria in terms of content and fully reflect the recommendations for initial diagnostic work up. Only the disease activity indices PCDAI for CD and PUCAI for UC were not included as variables in the CEDATA-GPGE registry during the study period, but can be calculated from other parameters using validated algorithms. Instead of the Disease Activity Index, the registry included the regular variable Physician overall assessment, which is used to provide a subjective assessment of disease activity. All variables are listed in Table 2. The right site of the table shows the 12 associated categories according to the Porto criteria.

**Table 2:** Identified variables of CEDATA-GPGE that reflect the Porto criteria

Diagnostic variables in CEDATA-GPGE	Category according to the Porto criteria
IBD in family	Case history
Condition	
Appetite	
Stool frequency during the day	
Stool frequency at night	
Blood in stool	
Stool consistency	
Abdominal pain during the day	
Abdominal pain during the night	
Abdominal finding	Physical examination
Anal finding	
Anal eczema	
Oral aphthae	
Cheilitis	
Extraintestinal manifestations	
Weight	Nutrition status
Height	Growth status
Breast development	Puberty stage^a^
Pubic hair	
Testicle volume	
Esophagogastroduodenoscopy Histology upper gastrointestinal tract	Endoscopy of the upper abdomen
Ileocolonoscopy	Ileocolonoscopy with multiple biopsies
Colonoscopy	
Histology lower gastrointestinal tract	
Diagnosis by	Diagnosis by pediatrician with gastroenterological expertise
Hematocrit	Initial laboratory investigations
Hemoglobin	
Mean corpuscular volume (MCV)	
Platelets	
Leukocytes	
Alanine aminotransferase (ALAT)	
Gamma-glutamyltransferase	
Albumin	
C-reactive-protein	
Blood cell sedimentation rate	
Magnetic Resonance Imaging—Enterography	Imaging of the small bowel^b^
X-ray in the small intestine	
Video capsule endoscopy	
Calprotectin	Stool samples
Physician overall assessment Disease activity index^c^	Disease Activity

^a^There are 3 variables regarding puberty, but only 2 of 3 variables per patient and gender can be filled in. ‘Pubic hair’ can be selected for both genders, ‘Breast development’ just for girls and ‘Testicle volume’ for boys

^b^Only 1 of 3 variables to be filled in per patient, because only one examination of these 3 is carried out—it depends on the type of imaging chosen

^c^No regular variable in CEDATA-GPGE, self-calculated from specific variables, which are basic for the determination of the disease activity index

### Documented examinations

Parameters of the category ‘Case history’ were documented for the largest part of the patients: For more than 80% of patients 7 out of 9 variables in this category were documented. In the category ‘Physical examination’, the parameters ‘Extraintestinal manifestations’ (93.6%) and ‘Abdominal findings’ (92.0%) were the most documented diagnostic measures. The category ‘Puberty stage’ was documented by the variables ‘Breast development’ (48.8%, only female), ‘Pubic hair’ (49.5%, female and male) and ‘Testicular volume’ (36.3%, only male). The least documented category was ‘Imaging of the small bowel’ with 39.1%. In the category ‘Disease activity’, the parameter ‘Physician's overall Assessment’ was assessed for 68.9% of the patients with CD, for 72.2% of patients with UC and for 67.4% of patients with IBD-U. The disease activity index could be calculated for 43.3% of patients with CD and for 76.9% of patients with UC. All categories are listed in Table 3.

**Table 3:** Measures documented in the diagnostic categories

Instrumental Diagnostic Category	Crohn ‘s Disease *n* = 289	Ulcerative Colitis *n* = 212	Unclassified IBD *n* = 46
	**%**		
Case history	79,9	76,4	73,9
Physical examination	74,1	76,6	69,6
Growth status	66,4	72,6	58,7
Nutrition status	48,4	47,6	47,8
Puberty stage^a^	74,9	59,7	53,2
Laboratory investigations	72,2	73,0	67,8
Stool samples	58,1	59,0	47,8
Ileocolonoscopy with multiple biopsies	41,1	43,4	42,0
Endoscopy of the upper abdomen	59,5	56,1	55,5
Imaging of the small bowel^b^	48,4	28,8	28,3
Overall medical assessment	68,9	72,2	67,4
Diagnosis by pediatric gastroenterologist	86,5	83,0	89,1
Disease activity index^c^	43,3	76,9	-

^a^There are 3 variables regarding puberty, but only 2 of 3 variables per patient and gender can be filled in

^b^Only 1 of 3 variables to be filled in per patient, because only one examination of these 3 is carried out—it depends on the type of imaging chosen

^c^Self-calculated from specific variables, which are basic for the determination of the disease activity index

For the categories ‘Case history’, ‘Physical examination’, ‘Nutrition status’, ‘Growth status’, ‘Laboratory investigations’, ‘Diagnosis by’, ‘Ileocolonoscopy with multiple biopsies’, ‘Endoscopy of the upper abdomen’, ‘Bone mineral status’ and ‘Stool tests’ there were no significant differences in frequencies between CD, UC, and IBD-U. Differences in frequencies existed in the category ‘Imaging of the small bowel’ (χ^2^ = 20.7, Cramer-V = 0.2, *p* < 0.001). In patients with CD, this category was documented more often than in patients with UC and IBD-U. The category ‘Puberty stage’ has significant differences in frequency as well (χ^2^ = 9.8, Cramer-V = 0.1, *p* < 0.05). It was documented in patients with CD more often than in patients with UC and IBD-U.

The diagnosis was confirmed by a pediatric gastroenterologist in more than 80% of patients in all three diseases (IBD-U: 89.1%, UC: 83.0%, CD: 86.8%).

The proportion of documented instrumental diagnostics showed an overall positive trend over the study period in all three categories ‘Endoscopy of the upper abdomen’, ‘Ileocolonoscopy with multiple biopsies’ and ‘Imaging of the small bowel’ (see Additional file 2). Especially the number of the reported ‘Esophagogastroduodenoscopy’ increased over all three diagnoses during the observation period.

#### VEO-IBD

According to the registry, only one child with CD was screened for immunodeficiencies in patients under 6 years of age (*n* = 29; 5.3%), which is relevant for differential diagnosis and to avoid treatment complications for this age group. No patients under 2 years of age (*n* = 5; 0.9%) were screened for immunodeficiencies and allergies as recommended by the Porto criteria. The questionnaire showed, however, that four of the five largest centers performed the examination in at least 75% of under 6-year-olds and that four of the five largest centers did so in 100% in under 2-year-olds.

### Supplementary survey

Each of the five centers contacted responded to the supplementary survey. On average, the centers treat 160 children and adolescents with IBD per year (min: 80, max: 300). On average, 78% of children and adolescents is in the age group > 10 to 18 years (min: 70%, max: 89%), 18% is between > 6 and 10 years of age (min: 9%, max: 25%) and 5% is up to 6 years of age (min: 4%, max: 5%). CD was diagnosed on average in 48% of children and adolescents (min: 40%, max: 62%), UC in 39% (min: 31%, max: 45%) and IBD-U in 13% (min: 8%, max: 20%). The proportion of under 6-years-old that is screened for immunodeficiencies is on average 72% (min: < 5%, max: 100%). The proportion of under 2-years-old that is screened for immunodeficiencies and allergies is on average 81% (min: < 5%, max: 100%). The proportion of patients with PIBD reported in the CEDATA-GPGE registry is in mean 54% (min: 20%, max: 70%). Reasons for not reporting every patient with PIBD include lack of staff and time in five centers and lack of consent form retrieval from patients in two centers.

## Discussion

### Reproduction of the Porto criteria in the registry

The registry fully reflects the guideline’s recommendations for the initial diagnosis of PIBD. The disease activity indices PCDAI for CD and PUCAI for UC were not included as variables in the CEDATA-GPGE registry during the study period. However, the PCDAI could be calculated in more than 75% of CD cases, while the PUCAI could be calculated in less than half of the UC cases. Through the variable ‘Physician overall assessment’, disease activity could be subjectively assessed by the treating physician as an alternative basis for decision making for further treatment. In 2016, the algorithms for calculating disease activity indices and thus the variables PCDAI and PUCAI could be implemented in the registry.

### Practical application of the guideline recommendations documented in the registry

The observed symptoms in our study sample corresponds to the typical triad of symptoms of PIBD reported in the literature [15, 24]. The proportion of documented diagnostic examinations varied within the 12 diagnostic categories and also between the three diagnoses. Some categories have a high level of documentation across the three diagnoses, e.g. ‘Case history’ and ‘Physical examination’. Some of the frequencies of the instrumental diagnosis categories, such as the ‘Imaging of the small bowel’, differ between documented diseases. *‘*Imaging of the small bowel’ and ‘Ileocolonoscopy with multiple biopsies’ are categories in which examinations are documented poorly for all three diseases. Furthermore, the very low documentation of ‘screening for immunodeficiencies’ in children and adolescents under 6 years of age is conspicuous. The supplementary survey indicated that while the majority of screening is performed, documentation in the registry is often missing. This suggests an underreporting, e.g., due to lack of capacity. Another possible reason is the effort involved in documentation. The documentation needs to be minimal and performed by dedicated personnel. This is further underlined by three phenomena: First, categories that are less relevant in the disease context tend to be less complete, e.g., growth retardation and pubertal delay are far more common in CD than in UC. This is also applicable for upper GI endoscopy and small bowel imaging, although the later may be delayed or at another institution depending on access to MRI or video capsule endoscopy. The second category involves high detail data input, e.g. laboratory values. This needs to be simplified by offering various entry methods (SI units or common other units) or automated transfer of data from hospital information systems (HIS), like evaluated in current and future projects. The last category is data with search effort in the registry, e.g. less common lab investigations like trough levels, vaccination status, or screening for immunodeficiencies. Our survey revealed, that far more of these investigations are performed than reported. This underreporting can only be addressed by improving data entry mechanisms and active query management for missing data and plausibility checks, as also addressed in current projects in CEDATA-GPGE. The registry has been intensely improved from 2016 on to address these issues, including live plausibility checks and improvement in data entry mechanisms. The increase in data documentation from 2016 on may be due to the Online-Tool 2.0. Automated or semiautomated data transfer from HIS is still not possible, but will be implemented in future projects in concurrence with rollout of the German Telematic infrastructure initiative.

The increase in instrumental diagnostics in all three diagnoses between 2014 and 2018 is in line with the observed increase in ileocolonoscopy and esophagogastroduodenoscopy by Buderus et al. for the period 2004 to 2014 [24]. Since 2011, the German Society for Gastroenterology, Digestive and Metabolic Diseases (DGVS) recommend esophagogastroduodenoscopy not only in CD but also in UC [33]. The Porto criteria recommend esophagogastroduodenoscopy in all suspected cases of IBD, too [17]. During our observation period, a lower rate of ileocolonoscopy and esophagogastroduodenoscopy was performed compared to Buderus et al. [24]. The low rates of some instrumental diagnostic measures, such as endoscopy, must be viewed with caution, considering input errors or incorrect reporting procedures or a diagnosis including endoscopy that took place outside the reporting center. The documentation characteristics in the registry have changed in 2016, as described above, which may lead to better documentation. This will be evaluated in future studies.

Which examination technique was used may depend on advances in medical technology and availability at a clinic or practice [34]. For example, X-ray was a common examination for imaging of the small bowel for a long time. However, because radiography has undesirable side effects (radiation exposure) for young patients, gentler examinations such as MRI or video capsule endoscopy should be preferred [17]. The data of Buderus et al. [24] as well as our study show that this recommendation finds acceptance among documenting centers.

The recommendation ‘Diagnosis by gastroenterologists with pediatric expertise’ is not in itself a diagnostic measure in the strict sense, but it is the most frequently documented variable from the recommended measures for diagnosis. This is due to the fact that mainly pediatric gastroenterologists document in the registry. Patients with IBD-U were diagnosed by a pediatric gastroenterologist more frequently than patients with CD or UC. It is conceivable that patients present to a specialist more often when findings are unclear, that is typical for IBD-U. IBD-U patients are younger and thus more prone to be treated by pediatric gastroenterological specialists.

The accurate documentation in the registry according to current guidelines is an important challenge for the participating centers but also for the administration of the CEDATA-GPGE registry. Missing documentation may influence the determination of important parameters, such as disease activity indices. An accurate documentation leads to improved process and outcome measures in children and adolescents with IBD, as Crandall et al. showed [23].

It should be considered that in some cases deviations from guidelines are justifiable [35]. Reasons may be disease type, age group, previous diseases, comorbidities, but also acceptance and reliability of parents. In practice, local availability is also likely to play a role. For example, by far not all outpatient clinics have access to video capsule endoscopy or easy access to MRI. The documentation of structural data of the reporting centers should be implemented in CEDATA-GPGE in the future in order to expand the possibilities of the registry for scientific questions of health services research. This kind of data allows comparisons between hospitals (benchmarking) [36] but also between the levels of care, staff and their qualification. Furthermore, they can provide insights into the use of certain equipment in the clinics [16, 34].

There may be reasons why some measures were not applied or documented in the registry, but these reasons were not documented in the registry during the observation period. Automated plausibility checks offer the opportunity to facilitate manual data input and to minimize missing data [26]. Since 2016, a new feature in the registry is that practitioners can use comments to inform about their decision on a specific issue. To increase data quality, regular data monitoring will be conducted so that individualized feedback can be provided to the recruiting centers [37]. Financing of dedicated personnel capacity for external quality assurance, as is mandatory by German law (§135a SGB V German Social Law), should be strictly implemented to ensure adequate participation and representative data extraction.

### Limitations

Generalizations or transfer of the results to the entire patient population with PIBD in German-speaking countries are not possible, because the documenting centers are selective compared to the overall treatment of PIBD. Only patients who were diagnosed in specialized centers and gave written consent were included in the registry. Other reasons why patients are not included in the registry may be time restrictions during data collection or in the centers; presentation in the emergency department or other clinics; change of clinic with loss to patient’s first reports; changes in the nursing, medical and scientific staff of an outpatient clinic. Four of the five centers of the additional survey stated, that they report between 50 and 70% of patients in the registry. Lack of staff and time were frequently mentioned as reasons for not reporting all patients. Therefore, the number of patients documented in the registry is smaller than the number who actually were diagnosed at the participating centers. Consequently, the registry is at risk of inclusion and exclusion bias, respectively, and there is a risk of overinterpretation of the results (selection bias). By choosing to survey the five largest centers rather than a random sample, it is likely that there is a selection bias here as well. A funnel plot was performed to check the relative contribution of each participating center to the results. This revealed heterogeneity, meaning larger centers included more patients, that may dominate the results.

### What implications can the results provide for health care research based on patient registries?

The aim of health care research is to generate knowledge about disease-specific diagnostic and therapeutic care, to evaluate treatment systems scientifically, and to derive recommendations for improvement that are relevant to patients or populations [38]. On the basis of patient-registries, the diagnostic of defined patient populations can be observed in a real healthcare setting [39]. Depending on the number of variables implemented, disease-related patient registries offer a very good overview of guideline-based care provided by participating centers. However, the data do not represent the entire reality of health care of PIBD. Disease-specific patient registries such as CEDATA-GPGE provide important insights into the practice of highly specialized care. Nonetheless, the majority of care for patients with PIBD in Germany and Austria is managed outside of the centers that participate in the registry. Combination with other data sources, such as comparison with reimbursement data from health insurance companies, can provide additional insights into care in the different sectors and levels of care in the healthcare system.

The German Network for Health Services Research (DNVF) named criteria for data quality in patient registries in 2010 (updated in 2019). The network emphasized that due to the predominantly missing legislative basis, sufficient registry quality (completeness and validity of the data) can only be achieved by a high acceptance of the registry by patients and documenting institutions [39, 40]. Incomplete and non-valid data are a major problem of patient registries. They often do not occur randomly and may contribute to result bias or false conclusions. Data quality highly depends on the administration of the registry in addition to the compliance of the documenting institutions [40].

Registries need a high administrative, temporal and financial effort [39]. The financing of the CEDATA-GPGE registry was realized by donations during the study period only. The longer a registry exists, the more experience is generated over time and implemented in the registry. Consequently, time itself is a quality-enhancing factor for registries in the sense of the quality improvement cycle (PDCA) according to Shewhart [36].

Patient registries can help health care communities to deal with three aspects of change over time. For once, there is mostly continuous but sometimes disruptive change in recommendations and therapeutic strategies with development of new treatments or better understanding of disease behavior. Besides capturing adherence to diagnostic and treatment recommendations, it may be valuable to obtain detailed case data to compare treatment strategies and adjust for captured confounders, e.g., with advanced models like propensity score matching instead of unreflective historical control groups that would lead to overestimation of treatment effects in current therapies [41].

Secondly, there is development of disease classification systems over time, which leads to differentiated therapeutic stratification. In registries, one can support this by applying new classification systems to previously documented cases with known follow-up and outcome. In PIBD, this is currently reflected by the definition of atypical UC but also the role of isolated Crohn’s colitis [42].

Finally, technological innovation leads to new investigation and data acquisition methods. Data acquisition may lead to improvement of data quality and especially completeness, but it does not do so by itself or automatically, as shown above. However, it can increase usability. It can incorporate other data sources, as currently observed with patient-reported outcomes through the CEDMO-app, that links patient diaries with CEDATA-GPGE registry data and help adolescents with IBD in everyday life [43]. It can also lead to the need for biobanking with patient registries to obtain biological samples that may be reexamined with new technologies later.

Disease-related patient registries can support the recruitment of patients for future studies in rare diseases, which in turn can provide important insights into guideline-based care. Clinical trials in comparison are much more restricted by inclusion and exclusion criteria, creating a homogenous dataset, that does not reflect real world application in most cases. In addition, registries have a longer observation interval than clinical trials, allowing the acceptance and application of current guidelines to be observed over a very long time. The additional implementation of patient related outcomes, e.g. quality of life, provides the opportunity to evaluate the effect of adherence to medical guidelines as implemented in CEDATA-GPGE recently [40].

## Conclusion

In conclusion, the Porto criteria for diagnostics in PIBD are fully represented in the registry CEDATA-GPGE. Therefore, the registry is a good basis for quality assurance in diagnostic and treatment of PIBD. The proportion of documented diagnostic examinations varies between the diagnostic categories and between the diagnoses CD, UC and IBD-U for the period from 2014 to 2018. It is important to note that not all performed diagnostic examinations are documented in the registry, e.g. due to lack of time or personnel. Therefore, sufficient time and personnel capacities are necessary to ensure reliable data entry and to enable researchers to derive important insights into guideline-based care.

## Supplementary Information

**Additional file 1.**

**Additional file 2.**

**Additional file 3.**

## Data Availability

The data that support the findings of this study are not publicly available. The data are available from the corresponding author upon reasonable request.

## Abbreviations

CD: Crohn’s disease
DNVF: German Network for Health Services Research
ESPGHAN: European Society of Pediatric Gastroenterology, Hepatology and Nutrition
GPGE: Society of Pediatric Gastroenterology and Nutrition
IBD: Inflammatory bowel disease
IBD-U: Unclassified inflammatory bowel disease
IQR: Interquartile range
PCDAI: Pediatric Crohn´s disease activity index
PIBD: Pediatric inflammatory bowel disease
PUCAI: Pediatric ulcerative colitis activity index
UC: Ulcerative colitis
VEO-IBD: Very early onset inflammatory bowel disease
